# Focusing on Future Applications and Current Challenges of Plant Derived Extracellular Vesicles

**DOI:** 10.3390/ph15060708

**Published:** 2022-06-03

**Authors:** Yuchen Wang, Junfeng Wang, Jinqian Ma, Yun Zhou, Rong Lu

**Affiliations:** 1 Marine College, Shandong University, No. 180 Wenhua West Road, Weihai 264209, China; 202017668@mail.sdu.edu.cn (Y.W.); 202017657@mail.sdu.edu.cn (J.M.); 202017686@mail.sdu.edu.cn (Y.Z.); 2 Newland Biotechnology Co., Ltd., No. 213 Huoju Road, Weihai 264200, China; junfeng.wang@neolandbio.com

**Keywords:** plant derived extracellular vesicles, separation, therapeutic application, drug delivery

## Abstract

Plant derived extracellular vesicles (EVs) are nano-sized membranous vesicles released by plant cells, which contain lipids, proteins, nucleic acids and specific pharmacologically active substances. They are safe, widely available and expediently extractive. They have gratifyingly biological activity against inflammation, cancer, bacteria and oxidative aging, especially for the prevention or treatment of colitis, cancer, alcoholic liver, and COVID-19. In addition, as natural drug carriers, plant derived EVs have the potential to target the delivery of small molecule drugs and nucleic acid through oral, transdermal, injection. With the above advantages, plant derived EVs are expected to have excellent strong competitiveness in clinical application or preventive health care products in the future. We comprehensively reviewed the latest separation methods and physical characterization techniques of plant derived EVs, summarized the application of them in disease prevention or treatment and as a new drug carrier, and analyzed the clinical application prospect of plant derived EVs as a new drug carrier in the future. Finally, the problems hindering the development of plant derived EVs at present and consideration of the standardized application of them are discussed.

## 1. Introduction

With the research and development of the active ingredients of natural drugs by scientists, active ingredients such as polysaccharides, saponins, phenols, terpenoids and flavonoids in plants have been extracted, analyzed and applied [1,2,3]. They have shown excellent therapeutic effects in most diseases. However, due to the single composition of these chemicals, their therapeutic effects in refractory diseases such as cancer and enteritis are not ideal. Although substances such as paclitaxel have good effects in a variety of cancer treatments, the side effects caused by their chemical properties limit their oral application [4]. However, plants, as a wide variety of drug sources, are a huge advantage over other animal derived therapeutic drugs. Animal exosomes have excelled in disease treatment. Plant derived EVs from a wide range of sources contain therapeutic chemical components and RNA components, which may be a promising natural source drug.

Since animal exosomes were first discovered in mammalian cells in 1983 [5], they have been extensively studied in different scientific departments. Exosomes are actively secreted by a variety of living cells to the extracellular space, and their size is 30–150 nm [6]. Their biological process is mainly divided into three stages: first, the cytoplasmic membrane sinks to form endocytic vesicles, and then fuses to form early endosomes; secondly, early endosomes entrap intracellular substances again, forming multiple intraluminal vesicles (ILVs), and further transform into multivesicular bodies (MVBs). Finally, MVBs fuse with a cytoplasmic membrane to release ILVs to the extracellular space, namely exosomes [7]. Exosomes carry proteins and nucleic acids involved in intercellular communication in various physiological and pathological processes [8]. Cells internalize exosomes via several routes: endocytosis, macro pinocytosis, phagocytosis, and membrane fusion [9]. At present, research on exosomes tends to mature, manifested in the drug delivery system, nutrition, clinical diagnosis and treatment [10,11]. However, there are certain limitations associated with exosomes, the most significant being the selection of the biological source for producing highly biocompatible exosomes on a large scale [12]. It would be better if a nanobubble with a wide range of sources and similar functions to animal exosomes could appear and be applied.

The fusion of MVBs and the cell membrane to release animal exosomes is the pathway of animal exosomes [7]. In 1967, MVBs were proved to exist in plant cells, so it is possible for plant derived EVs to be released through the fusion of MVBs and the plasma membrane. In a transmission electron microscopic study of barley leaf cells invaded by fungi, An et al. clearly observed plant MVBs [13]. In 2007, researchers confirmed that the fusion of MVBs and the plasma membrane in higher plants may lead to the release of EVs into the extracellular space [14]. In fact, a few studies have proved that MVBs mediates the secretion of exosomes in plants [15]. The protein identified by Eric Woith et al. shows that the mechanism of plant cells secreting EVs is similar to that of animal cells releasing exosomes and microcapsules [16]. Although the exact mechanism of plant derived EVs is unclear, some studies have shown that the possible sources of plant derived EVs include exocyst positives organelles (EXPOs), MVBs, vacuoles, and autophagosomes [17,18]. In 2013, the Huang-Ge Zhang team successfully extracted EVs from grapes for the first time with reference to the isolation methods of animal exosomes. Although these grape derived EVs were not identical to animal exosomes, they shared HSP70 and aquaporin, were rich in phosphatidic acid (PA) and phosphatidylethanolamine (PE) lipids and were similar in structure and composition [19]. With the in-depth study of plant derived EVs, EVs derived from a variety of fruits and vegetables have been isolated, some even from traditional herbs and fungi [20].

In recent years, researchers have gradually found and isolated EVs with particle sizes in the range 30~500 nm from plants and characterized them [19]. Compared with animal exosomes, plant derived EVs have a larger particle size range. Plant derived EVs are nano-sized membrane vesicles isolated from fresh plant juice, which contain lipids, proteins, nucleic acids and other small molecular components [21]. Although plant derived EVs and animal exosomes contain proteins, lipids and RNAs, there is a clear difference between the two components. The differences between animal exosomes and plant derived EVs are shown in Table 1. These plant derived EVs play an important role in interkingdom communication, which contributes to plant growth and development, defense responses, and symbiotic relationships between plants and microorganisms [22,23]. In addition, plant derived EVs have diverse biological functions that generally correlate with the effects of the plant of origin. Recent data suggest that plant derived EVs may play an important role in maintaining intestinal symbiosis and internal environmental stability, such as ginger and broccoli derived EVs, which have powerful anti-inflammatory effects [24]. In addition, plant derived EVs have different targeting characteristics and the advantage of being derived from edible plants, thus avoiding the limitations of chemically synthesized nano-carriers in drug delivery: human toxicity or immunogenicity; limited target tissue uptake efficiency; potential adverse environmental effects; and high cost for large-scale production of nanomaterials [25,26,27]. Plant derived EVs have several advantages as vehicles for the treatment of diseases relative to synthetic nanocarriers. They show relatively high internalization rates, lower biological toxicity and immunogenicity. They are stable within the gastrointestinal tract and have the ability to cross the blood–brain barrier, but not the placental barrier. In addition, they can be produced economically on a large scale and can deliver many different types of drugs at the target site and there is efficient absorption [28]. Although the advantages of plant derived EVs in drug delivery, nutrition and treatment are relatively more potential, they lack standard rules for separation and application, in addition to extraction methods for both terms of extraction rate, purity, convenience and economy.

We comprehensively reviewed the latest separation methods and physical characterization techniques of plant derived EVs, summarized the application of them in disease prevention or treatment and as a new drug carrier, and analyzed the clinical application prospect of plant derived EVs as a new drug carrier in the future. Finally, the problems hindering the development of plant derived EVs at present and consideration of standardized application of them are discussed.

## 2. Composition of Plant Derived EVs

### 2.1. Structure of Plant Derived EVs

**Figure 1:**
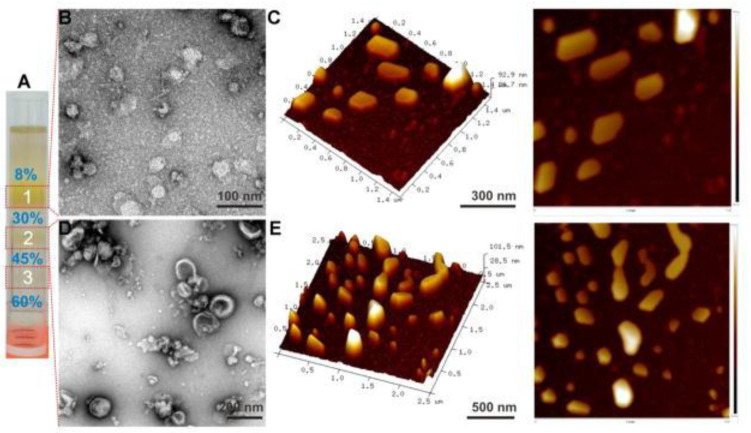
Ginger derived EVs. Sucrose gradient ultracentrifugation depicts three bands (**A**). Visualization and characterization of ginger derived EVs using Transmission Electron Microscope (TEM) (**B**,**D**) and atomic force microscope (AFM) (**C**,**E**) are shown. Reproduced with permission from ref. [33].

The structural characteristics of plant derived EVs are similar to those of animal exosomes. They are membrane structures with lipid bilayers, membrane proteins on the surface and proteins, nucleic acids and other substances in the interior (Figure 1). Their particle size is generally slightly larger than that of animal exosomes [10]. For example, the average diameter of ginger derived EVs is 284.1 nm and Zeta potential is −29.7 mV [29]. The average particle size of grape derived EVs is in 380.5 ± 37.47 nm, and the zeta potential value was negative, with an average potential of −26.3 ± 8.14 mV, showing good stability [19]. The particle size of EVs in lemon juice is 50–70 nm. The peak diameter of coconut water derived EVs is 138.1 nm, and the range of particle size is similar to that of grape derived EVs, which is significantly larger than that of milk exosomes and fat exosomes [30]. The size range of strawberry derived EVs is 30–191 nm [31]. The diameter of apple derived EVs is in the range 100–400 nm, and the average diameter is 170 nm [32]. These Nanovesicles generally have a small size range, between 30 nm and 500 nm, and generally have a negative Zate potential, which is above −20 mV, with high stability.

### 2.2. Composition of Plant Derived EVs

**Figure 2:**
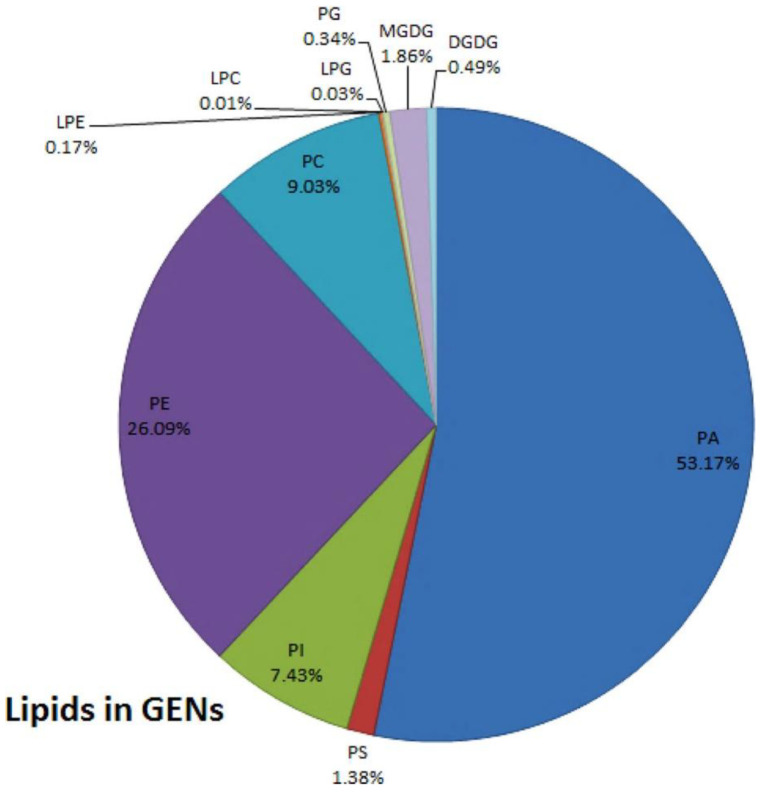
Synopsis of the putative lipid species in grape derived EVs; reported as percent of total grape derived EVs lipids, attained by sucrose gradient ultracentrifugation and differential centrifugation: LPC, lyso-phosphatidylcholines; LPE, lyso-phosphatidylethanolamines; LPG, lyso-phosphatidylglycerol; MGDG, monoglycerol; DGDG, diglycerol; PA, phosphatidic acids; PS, phosphatidylserine; PI, phosphatidylinositol; PE, phosphatidylethanolamines; PC, phosphatidylcholines; PG, phosphatidylglycerol. Reproduced with permission from ref. [35].

Lipids isolated from plant derived EVs extraction subjected to lipid profiling suggested that the two major lipid classes of plant derived EVs are phospholipids and glycerolipids, which readily accumulate in the organic phase layer upon classical liquid–liquid extraction (use Bligh and Dyer) [28]. Plant derived EVs are rich in lipids, including phosphatidic acids (PA), phosphatidylcholine (PC), phosphatidylethanolamines (PE) and so on [28] (Figure 2). The composition and content of lipids in EVs from different sources are distinct. The PA in ginger derived EVs accounts for about 35.2%, while the PA in grapefruit and garlic derived EVs only accounts for 3.5% and 5.5%. On the contrary, most of the lipids in grapefruit and garlic derived EVs are PC, which are 36.2% and 52.6%, respectively [34]. In grape derived EVs, PA accounts for 53.17%; PE accounts for 26.09%; PC accounts for 9.03%; and PI accounts for 7.43%. They also include some other lipids, but the content in EVs is very little. Additionally, the PA in grape derived EVs is enriched compared with the whole group of grape lipids [19].

Some channel proteins, enzyme proteins and heat shock proteins (HSP) are present on the membrane surface or in the membrane of plant derived EVs. At present, there are relatively few studies on the content and function of proteins in plant derived EVs. Therefore, there is no mature understanding of the protein components in plant derived EVs and the rules that can be followed. Through proteomics analysis of plant derived EVs, most of the homologous proteins of highly expressed proteins have been reported in animal exosomes. The protein contents of plant derived EVs from different plants are quite different. For instance, 56.7% of proteins from lemon derived EVs overlapped with those previously identified as exosome proteins in mammalian tissues [36,37]. Arabidopsis thaliana derived EVs are highly rich in proteins involved in biotic and abiotic stress responses. In addition, the content of transporters is also relatively abundant, and these protein components are enriched compared with the whole proteome of Arabidopsis thaliana [38]. Mass spectrometer analysis shows that grapefruit and grape derived EVs contain proteins that regulate carbohydrate or lipid metabolism, and grape derived EVs also have some channel proteins [29,39]. EVs derived from ginger contain less protein, mainly cytoplasmic protein, including actin and proteolytic enzyme. In addition, there are a few membrane proteins related to transport [40]. Although proteins from different plant derived EVs are not exactly the same, there are still some proteins in most plant derived EVs. For instance, citrus derived EVs [41], ginger derived EVs [33] and grape derived EVs [19] all contain reticulin heavy chain, HSP and aquaporin, etc. GTPase Rab was found in sunflower derived EVs [41]. Three exosome marker proteins were highly expressed in wheat derived EVs: HSP 70 (79.97%), CD9 (93.96%), and CD63 (86.39%) [42]. However, the protein content of EVs from lemon is slightly higher [36].The proteins in plant derived EVs include not only channel proteins, hydrolyzed proteins, but also proteins that play a regulatory role in the cytoplasm. 

In addition to lipids and proteins, plant derived EVs are also rich in RNAs, mainly MicroRNAs (miRNAs). The types and functions of these RNAs in EVs from different plants are different. Grape derived EVs contain miRNAs components, which mainly belong to the miR169 family [19]. There are extracellular miRNAs in coconut water, and the content of miRNAs in mature coconut water is higher than that in immature coconut water [43]. Other researchers specifically analyzed the EVs of 11 plant sources, including tomato, soybean, orange, pea, kiwifruit, cantaloupe, grapefruit, ginger, coconut milk and blueberry. A total of 418 miRNAs were identified from 11 edible plant derived EVs samples. Target prediction and functional analysis showed that the high expression of miRNAs was closely related to inflammatory response and tumor-related pathways. According to their plant derived EVs distribution, 418 miRNAs can be divided into three categories: 26 “frequent” miRNAs (FMs), 39 “moderate presence” miRNAs (MPMs) and 353 “rare” miRNAs (RMS). Compared with RMS, FMS had fewer kinds of miRNA, but its cumulative expression level is significantly higher [44]. MiRNAs in plant derived EVs have the potential to regulate mammal RNA [45]. 

In addition to proteins, nucleic acids, lipids and other common contents, plant-specific active components are also included in plant derived EVs. Man et al. studied the active components in ginger derived EVs and showed that the ginger derived EVs contained 6-gingerol, 8-gingerol and 10-gingerol. Compared with the same quality of ginger derived EVs and ginger, the content of gingerol in vesicles was much higher than that in ginger. These gingerols can be delivered from the vesicles to the intestines of rats and absorbed [46]. Perut et al. detected rich vitamin C in EVs derived from strawberries, showing a strong antioxidant effect [31].

## 3. Isolate and Characterize Plant Derived EVs

There are many kinds of vesicles in plant cells. Their particle sizes overlap, and their shapes are very similar. Therefore, it is very important to separate them from plant tissue relatively accurately in a simple and effective way. Because the structure of animal exosomes is similar to that of plant derived EVs, the isolation of animal exosomes is of great significance for the isolation of plant derived EVs. However, due to the difference between animal exosomes and plant derived EVs, the isolation of plant derived EVs needs to be improved. At present, there are a variety of methods to isolate plant derived EVs, including differential ultracentrifugation (UC) combined with sucrose gradient density centrifugation, PEG precipitation, size exclusion chromatography (SEC), kit, ultrafiltration membrane separation and so on. However, none of these methods are perfect in their applicability, extraction rate, extraction time and purity. These methods have their own advantages and disadvantages (Table 2). The combination of various methods may be a better option.

### 3.1. Ultracentrifugation

**Figure 3:**
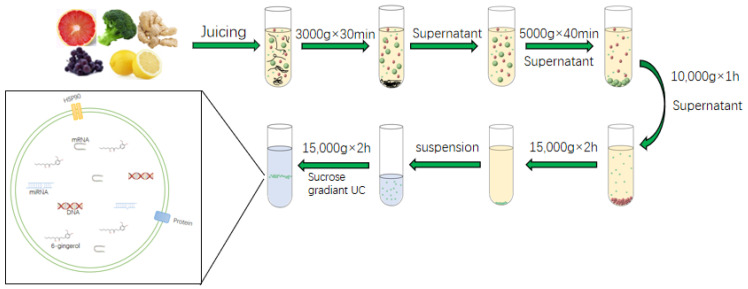
Isolation and Preparation of plant derived EVs.

Among the isolation of plant derived EVs, the most commonly used extraction method is UC. Then, the crude plant derived EVs are purified by sucrose gradient density centrifugation. Based on the different centrifugal force and sucrose gradient, different types of nanoparticles can be separated [28]. The general step of UC is to first wash the fresh fruit purchased from the market or picked by hand with PBS or tap water. Subsequently, the juice is ground in a blender and diluted with appropriate amount of PBS as needed; then, in the centrifuge, the supernatant is first centrifuged at a low speed to remove fiber debris in plant tissue, and then the centrifugation speed is gradually increased to remove smaller particles while retaining plant derived EVs in the supernatant. Of course, with the increase in the number of times, not only does the centrifugation speed gradually increase, but also the centrifugation time becomes longer and longer. After centrifuging at a relatively low speed for three times, the supernatant is retained. At this time, a centrifugal force of about 100,000–150,000 g was selected for 1.5–2 h. The precipitation containing plant derived EVs is recovered to remove the interference of other complex intracellular vesicles (Figure 3). The precipitate obtained is still resuspended with PBS, and then purified by sucrose concentration gradient centrifugation. Generally, the sucrose concentration gradient is 8%/15%/30%/45%/60%, and the EVs with high purity are obtained by removing the interference of other complex proteins, DNA and RNA. The portion between 30% and 45% are recovered and marked as plant derived EVs. A protein kit is used to quantify plant derived EVs according to protein content [40,47,48]. The purity of plant derived EVs obtained by this method is high, but it takes a long time and is highly dependent on the instrument [49]. The low extraction efficiency of this method and the possible aggregation of plant derived EVs that may affect the downstream functional analysis have led scientists to further explore ways to solve the above problems. Phat duong et al. found that the use of buffer density gradient ultracentrifugation and the use of a high-density iodosanol pad in the initial concentration stage in the extraction of plant derived EVs from macrophages can increase the yield of plant derived EVs and avoid aggravating protein pollution [50]. Therefore, perhaps the isolation of plant derived EVs can also be performed with a similar buffer or with the addition of a small pad to increase extraction efficiency. The above method for obtaining plant derived EVs has the characteristics of simple operation. The obtained plant derived EVs have higher purity and more particles. At present, this method is often used in the laboratory to separate plant derived EVs. However, this method is highly dependent on the requirements of ultra-high speed centrifugal force, and the use conditions of ultra-high-speed centrifuge are relatively strict. The most unsatisfactory is that the obtained plant derived EVs have a strong precipitation aggregation force and are difficult to fully disperse. Therefore, this method also needs to be optimized for more convenient use.

### 3.2. PEG Precipitation

Because the UC requires the use of high-cost ultra-high-speed centrifuge, the premise of mass production of plant derived EVs is to obtain a more economical and effective method without affecting the extraction quality. Based on the safety of PEG, PEG precipitation was invented by researchers. PEG precipitation and UC have the same steps of removing impurities in fruit juice through low centrifugal force in the early stage. Obtaining the plant derived EVs in the later stage is different. In the PEG precipitation, an appropriate amount of PEG-6000 was added to the supernatant, shaken overnight at 4 °C. Then, centrifuged for 30 min with low centrifugal force the next day, and re-suspended with PBS. The extraction rate of PEG precipitation is close to that of UC, which is 60–90% of that of overspeed differential centrifugation. By characterization, the average diameter of plant derived EVs obtained by this method is slightly smaller than that of UC. The zeta potential value is similar, and the composition of RNA, protein and lipid is similar. In addition, they can also be absorbed by cells [51]. Through a series of changes in pH in sedimentation conditions, the best extraction effect is obtained when pH is 5. The amount of extraction is not only increased, but also its physical properties hardly change [52]. Although plant derived EVs obtained by PEG precipitation are similar to those obtained by UC, further optimization is needed if they are to be widely used, but it has to be said that PEG precipitation has the potential to produce plant derived EVs economically on a large scale. To sum up, compared with UC, this is a simple and convenient method of operation. However, the purity of plant derived EVs obtained by this method is low. Due to the influence of complex components in plant juice, PEG forms a grid to capture plant derived EVs while capturing other impurities in juice, such as negatively charged free proteins in juice [53]. The low purity and high cost limit the application of PEG precipitation. Plant derived EVs with higher purity can be obtained if the obtained precipitate is purified by the sucrose density gradient centrifugation method.

### 3.3. Size Exclusion Chromatography

SEC is also used by researchers to isolate plant derived EVs, but it is rarely used. SEC is based on the molecular sieve effect on the selectivity of different molecular volumes in the separation of different molecules through the column filler, as speed through pores differs to achieve the purpose of separation. The method of isolating plant derived EVs is to first squeeze the plant into juice, and then low-speed centrifugation to retain the supernatant to remove larger plant fiber debris, etc. Then, the supernatant was separated by SEC chromatography. Because the size of the plant derived EVs is generally larger than that of the impurity protein, the EVs would flow out through the chromatographic column at first, while the impurity protein particle size is smaller and remains in the chromatographic column for a longer time. Thus, different components are obtained at different times, and the separation of plant derived EVs, and impurity proteins is realized. You JY et al. [54] used SEC, UC and PEG precipitation to isolate cabbage derived EVs. The cabbage derived EVs obtained by these three methods are characterized and their purity is determined. It is found that compared with the other two methods, the EVs obtained by SEC have a more uniform particle size and higher extraction rate per unit mass of plants. The purity is significantly higher than that of the EVs obtained by two other methods. However, the separation may take longer than the two other methods. This method is mainly used to separate impurity proteins and plant derived EVs, and the obtained plant derived EVs can maintain good biological activity. However, the preparation process is cumbersome and time-consuming, and it is difficult to separate large-size impurity particles.

### 3.4. Electrophoresis

The separation of Plant derived EVs by electrophoresis combined with dialysis is mainly based on the fact that under the action of the electric field, the charged proteins and RNAs will move to the positive and negative poles, and they can be enriched on both sides of the electrode through the dialysis bag. Additionally, plant derived EVs will continue to stay in the dialysis bag, so as to achieve the purpose of separation. Meng Yang et al. [49] found a new separation method. They used the electrophoresis technique combined with 300 kDa cut-off dialysis bag (ELD) to separate lemon derived EVs. The dialysis bag containing lemon juice was placed into the electrophoresis device and the power supply was turned on and connected to the positive and negative electrodes. Under the action of the electric field, RNA and protein can enter the electrophoresis buffer through the dialysis bag, and the EVs become trapped in the dialysis bag. After 2.5 h, lemon derived EVs are obtained. This method saves time and does not require special equipment. The size and number of EVs isolated from lemon by the ELD technique are similar to those isolated by UC.

### 3.5. Other Methods

There are some articles showing that researchers use commercially available separation kits to separate plant derived EVs [55]. This method is simple to operate, but the separation effect is poor. Some proteins of similar size and various impurities are too different to be effectively separated, thus affecting the separation effect of plant derived EVs. The number of each separation can also be small and therefore unsuitable for large-scale production.

Due to the advantages and limitations of each method, researchers obtained higher quality plant derived EVs by combining the above separation methods. Ultrafiltration is usually used as a combination of other methods to separate plant derived EVs. Ultrafiltration depends on the use of external driving forces to drive smaller molecules through specific pore size polymer membranes, while retaining larger molecules [56]. Some practices have proved that the combination of differential centrifugation and ultrafiltration is an economical separation method. Lee et al. used a 100 K centrifugal filter to gather EVs from the stem and leaf juice of Dendropanax morbifera after differential centrifugation [57]. This method first removes impurities such as large fiber fragments in juice by medium speed centrifugation, and then removes impurities by ultrafiltration, avoiding the use of highspeed centrifugal equipment, which has the advantages of cost-effectiveness and commercial practicability [58,59]. However, since the particle size of impurity proteins is usually smaller than that of our target particles, the purity of the sample needs to be confirmed.

### 3.6. Physical Characterization

Plant derived EVs obtained by the above methods require further measuring of their particle size, morphology, zeta potential and so on to determine the authenticity and stability of the particles. Most researchers first measure the size distribution of plant derived EVs by dynamic light scattering (DLS) [60]. The particle size distribution of plant derived EVs is related to the intensity of light scattered by each particle [43]. The morphology and size of fine particles were observed by TEM and AFM [60]. Scanning electron microscope (SEM) is also a common instrument for observing the three-dimensional morphology of plant derived EVs. Before observation, a series of operations need to be carried out, such as immobilization with glutaraldehyde or 1% uranylacetate, dehydration of ethanol and so on [40,43]. This instrument has the advantages of continuous and tunable amplification effect, large field of view, and good imaging stereo effect. In addition to the above, nanoparticles reconstituted with plant derived EVs derived lipids could also be examined for size and morphology by the above methods [61].

## 4. Bioactivity of PELVNs

It has taken a long time for plants to be used to treat diseases, with native forms or extract active substances such as polysaccharides, phenols and terpenoids used to inhibit the development of diseases and repair the damage caused by diseases. However, research on plant derived EVs, a new plant component, has only begun in recent years. After preliminary exploration by researchers, plant derived EVs from various edible plants have been shown to have good biological activities, such as anti-inflammation, anti-cancer, anti-bacteria and fungi, and anti-oxidation. They have excellent effects through a variety of pathways, such as gene regulation, intestinal flora, macrophages, gene silencing and their own specific active molecules (Table 3).

### 4.1. Anti-Inflammatory Effect

Inflammation is generally regarded as a phenomenon caused by the imbalance of immune dynamic balance. If this imbalance is not controlled, it may develop into acute or chronic inflammatory diseases, affecting people’s health [62]. Some plant derived EVs, such as from ginger, grapefruit and grape, have shown their superior anti-inflammatory effects in relevant studies [19,29,40]. Studies in this area have shown that both lipids and RNA in plant derived EVs may play a role in anti-inflammation, and the difference between them lies in the mechanism of anti-inflammation [19,34,48]. Lipid components play a very important role in the anti-inflammatory effect of plant derived EVs. They may treat inflammation by regulating the gene expression of cells in inflammatory parts of the body, and they can also improve inflammation by acting on macrophages [29]. In addition to these, a number of miRNAs are included. These RNAs, which are able to change the composition and physiology of the intestinal flora, can be taken up first by the microbiota in the gut. Then, they affect the transcription and translation of RNAs, and change the number of downstream inflammatory factors or chemokines to achieve the effect of reducing inflammation. The anti-inflammatory effect of plant derived EVs is particularly prominent in the treatment of acute and chronic colitis. Ginger derived EVs strongly induce macrophages to express HO-1 and IL-10 and exert antioxidant and anti-inflammatory effects [48]. Ginger derived EVs can also rely on the mechanism of IL-22 production to improve colitis in mice. After entering the intestine, Ginger derived EVs were first ingested by lactobacilli in a lipid dependent manner because it was rich in PA. Mdo-miR7267-3p in Ginger derived EVs mediates the targeting of LGG monooxygenase ycnE and up-regulates the amount of I3A. I3A is the ligand of aromatic hydrocarbon receptor (AHR) to induce the production of IL-22 and inhibit the induction of proinflammatory cytokines IL-1 β and TNF α. IL-22 enhances the defense ability of the barrier and alleviates the symptoms of colitis [34]. Plant derived EVs, including grape and grapefruit, can induce Wnt/TCF4 activation [48]. After oral administration of grape derived EVs, they target intestinal stem cells and promote the proliferation of BMI1 stem cells; the gene expression of SOX2, Naong, OCT4 and KLF4, a marker of pluripotent stem cells, was significantly up-regulated; and it could also trigger downstream normative Wnt signal activation, induce Lgr5+ stem cell proliferation, accelerate intestinal mucosal regeneration, promote intestinal tissue remodeling, protect the intestine of mice and inhibit DSS-induced colitis [19,48]. In summary, ginger derived EVs play an anti-inflammatory role through the activation of Nrf nuclear translocation and the induction of anti-inflammatory cytokines. Grapefruit derived EVs play a leading role in anti-inflammatory regulation of Wnt. Nrf2 nuclear translocation and Wnt/TCF4 activation play a key role in anti-inflammatory response [48,63,64,65].

### 4.2. Anticancer Effect

Cancer has a long history. Many methods and drugs have been found to fight against malignant tumors. However, due to the stubbornness of malignant tumors and the side effects of drugs, we have not found the best of both worlds. Although there are many drugs that can treat cancer, their non-specific effects on the immune system can lead to short-term and long-term debilitating side effects, such as allergic reactions, nausea, elevated liver function, pancreatitis and other life-threatening side effects. Further application is limited [66]. At the same time, plant derived EVs have little side effects and show varying degrees of therapeutic effects on many types of cancer. According to the work carried out by the researchers, plant derived EVs such as from citrus lemon, ginger, grape, grapefruit and Chinese bamboo shoots all have anti-cancer effects [67,68]. Lemon derived EVs has anti-proliferation effect in vivo and in vitro. They can up-regulate GADD45a through ROS produced by tumor tissue and lead to S-phase arrest and apoptosis of gastric cancer cells [49]. EVs from citrus, lemon and grapefruit inhibit the growth of A375 (melanoma), A549 (lung adenocarcinoma) and MCF-7 (breast cancer) cell lines to varying degrees [69]. Tea flower derived EVs after intravenous injection or oral administration could accumulate in breast tumors and lung metastatic sites, inhibit the growth and metastasis of breast cancer, and modulate gut microbiota [70]. In addition, lemon derived EVs can also inhibit chronic myeloid leukemia in vivo by specifically reaching the tumor site and activating the TRAIL-mediated apoptotic cell process [36]. Asparagus cochinchinensis derived EVs have specific anti-proliferative activity against hepatocellular carcinoma cells and are related to the pathway of inducing apoptosis [68]. The safety, effectiveness, targeting and anti-proliferation of EVs open a new channel for the prevention and treatment of cancer.

### 4.3. Effects on Bacteria and Fungi

Research on the internalization of plant derived EVs and their effects on cell function has been very rapid, but the effects on bacterial growth need to be further studied and summarized. Plant derived EVs can promote some probiotics; on the contrary, they can inhibit the bacteria that are not conducive to the healthy development of the human body. Current studies have shown that some plant derived EVs can be absorbed by bacteria after co-incubation with bacteria under suitable conditions [71]. A possible mechanism of post-harvest regulation of bacterial growth is that internal miRNAs induces gene expression in bacteria. For example, EVs in coconut water can promote the growth of probiotics MG1655 and Lactobacillus plantarum WCFS1 [30]. Arabidopsis thaliana derived EVs can carry sRNA to the infected site and be absorbed by the fungal pathogen Botrytis cinerea. These sRNA can induce the silencing of key fungal genes [72]. Ginger derived EVs are selectively absorbed by Porphyromonas gingivalis in a ginger derived EVs-dependent manner by interacting with heme-binding proteins on the surface of Porphyromonas gingivalis. When ginger derived EVs bind to HBP35, the pathogenic mechanism of Pseudomonas gingivalis is significantly weakened after the interaction between Pseudomonas gingivalis and ginger derived EVs cargo molecules, including PA and miRNAs [71]. EVs with antifungal activity released from plant roots [73]. With the excellent performance of plant derived EVs in affecting the growth of bacteria, plant derived EVs also have good development prospects in the treatment of diseases caused by bacteria.

### 4.4. Antioxidation

Oxidation is the cause of aging, inflammation and other negative factors. The lipid bilayer vesicle structure of plant derived EVs can protect unstable antioxidants in the vesicles. The antioxidants in fruits and vegetables with significant antioxidant activity can be encapsulated in the corresponding EVs and transported to the plasmids in vitro. Lemon derived EVs are found to be rich in citric acid and vitamin C, which have a significant protective effect on the oxidative stress of mesenchymal stem cells (MSC) [74]. EVs derived from strawberries can be internalized by MSC without affecting their activity. The experimental results show that the EVs of strawberries can prevent oxidative stress in a dose-dependent manner, which may be due to the rich vitamin C in the vesicles [31]. The antioxidant effect of plant derived EVs has great potential in cosmetology and medicine. It is important to consider these plant derived vesicles as new ingredients in our food to evaluate their health benefits and the potential of food derived technologies.

## 5. Application of Plant Derived EVs in Human Diseases

People are very interested in whether plant derived EVs can play a role in interspecies communication and have direct benefits to human diseases [47,48,75]. The origin of lant derived EVs contain proteins, RNA, lipids and corresponding active ingredients, which can be absorbed in the intestinal tract by oral administration [46]. Additionally, they are then transported to different parts. Plant derived EVs also contain plant homologous components, so they have potential biological functions of homologous plants. Plant derived EVs can resist the extremely acidic environment in the stomach and the highly active proteolytic enzymes in the intestine [76]. Related studies have shown that edible plant derived EVs can be absorbed in the intestinal tract by oral administration, by regulating intestinal flora, or by inflammatory factors or affecting intestinal stem cells, or by sRNA regulating downstream gene expression [34,40]. According to the researchers’ application of plant derived EVs in animal models, EVs from different sources can play a role in various ways, such as oral administration, intravenous injection, nasal administration and transdermal administration. For safety reasons, oral administration is a relatively safe administration route. Plant derived EVs usually choose the appropriate administration route according to the needs of the disease. At the same time, EVs from the same plant source can also be administered in various ways to treat diseases (Table 4). They play a safer role in the treatment of animal models than drugs with strong side effects, such as the treatment of colitis, the treatment of alcoholic liver and so on.

### 5.1. Plant Derived EVs in the Treatment of Multiple Diseases

sRNA is a non-coding regulatory RNA with a length of about 20–30 nt. It is an important regulator of gene expression. It can silence genes with complementary sequences [45,77]. In animals, sRNA can be transported through EVs, specific transmembrane proteins, high density lipoprotein complexes or gap junctions [78]. Within plants, sRNA may move from one cell to another through plasmodesmata and systematically through the vascular system [79]. However, more and more evidence shows that sRNAs can be transferred not only in the cells and tissues of a single organism, but also across different eukaryotic species, as a link between the animal, plant and microbial world [45]. It has been proved that there are many kinds of sRNA in plant derived EVs. These sRNAs can be carried and transported into eukaryotic cells by plant derived EVs, and the corresponding therapeutic effect can be produced by RNA interference [34,72,80].

#### 5.1.1. Plant Derived EVs in the Treatment of Colitis

Colitis refers to the pathological changes in the colon caused by immune factors, genetic factors, environmental factors, infectious factors and other factors. The main clinical manifestations of colitis are abdominal pain, diarrhea, hematochezia and constipation for several days. Traditional treatment strategies rely on the frequent use of high-dose drugs, including antibiotics, non-steroidal anti-inflammatory drugs, biological agents and immunomodulators to reduce inflammation. Some of these drugs are effective in relieving early inflammatory symptoms, but their long-term efficacy is affected by cumulative toxicity [81]. Other nano-preparations that have entered clinical trials have failed due to early drug release, weak targeting and high immunotoxicity of their materials [82]. Therefore, it is still urgently needed to find a reasonable, safe, effective and stable nano-drug delivery system. Based on this, plant derived EVs have become a good choice due to their low immunogenicity, high stability and targeting. At present, many studies on plant derived EVs have shown high safety and effectiveness in the treatment of colitis [83]. Edible plant derived EVs may hold potential as new agents for the prevention and treatment of gut-related inflammatory disease. The lipid and RNA components of EVs from different sources are different, which also leads to differences in their cell specificity and mechanism of action [47]. According to different mechanisms of action, they are mainly divided into the following categories:

I. Regulating the function of intestinal macrophages.

Intestinal macrophages play an important role in maintaining mucosal tolerance and inhibiting inflammation in order to maintain the stable state of the host. However, under the pathophysiological conditions of inflammatory bowel disease (IBD), intestinal macrophages lose tolerance, resulting in uncontrolled intestinal inflammation. Therefore, regulating the function of intestinal macrophages is considered to be a very important strategy for the treatment of IBD patients [29].

Studies have shown that grapefruit derived EVs can be selectively absorbed by intestinal macrophages to improve dextran sulfate-induced colitis in mice. Intestinal macrophages uptake grapefruit derived EVs, up-regulate the expression of HO-1, and inhibit the production of IL-1 β and TNF- α in intestinal macrophages. In addition, due to the edible plant origin of grapefruit, its excellent biocompatibility and biodegradability, its stability in a wide range of pH and the targeting of intestinal macrophages, grapefruit- derived EVs have stronger safety compared with immunosuppressants and steroids, which have more side effects. Grapefruit derived EVs show high advantages as a potential drug or delivery system for the treatment of colitis. In terms of drug delivery, compared with free methotrexate (MTX), the incorporation of anti-inflammatory drug MTX into grapefruit derived EVs and delivery of MTX-Grapefruit derived EVs to mice significantly reduced the toxicity of MTX and significantly increased its therapeutic effect in DSS-induced colitis in mice. These findings suggest that grapefruit derived EVs can act as an immunomodulator in the gut to maintain intestinal macrophage homeostasis and can be developed for the oral delivery of small molecular drugs to reduce inflammation in human diseases. Therefore, as a new drug delivery system, grapefruit derived EVs can be taken orally to reduce human inflammatory response in diseases [29].

II. Promote the proliferation of intestinal stem cells.

Grape derived EVs can protect mouse colitis induced by DSS by promoting intestinal Lgr5hi stem cell proliferation and mediating intestinal tissue remodeling in response to pathological triggers. After oral administration of grape derived EVs into the intestinal tract, the fluorescence signal showed that it accumulated in the intestinal tract within the first 6 h, and then gradually decreased but was not completely cleared within 48 h. Grape derived EVs targeted Lgr5hi mouse intestinal stem cells and promoted their proliferation, accelerating mucosal epithelial regeneration. The mixture is stronger. The research mechanism found that after grape derived EVs were ingested by intestinal stem cells, the number of BMI1 genes was increased; the gene expression of stem cell marker SOX2, Naong, OCT4, KLF4 was significantly up-regulated by encoding pluripotent stem cells; in addition, it triggered downstream normative Wnt signal activation, induced Lgr5+ stem cell proliferation, accelerated intestinal mucosal regeneration, promoted intestinal remodeling, protected mouse intestine and inhibited DSS-induced colitis. Among them, the LLN assembled by grape derived EVs lipids showed the function of targeting and inducing the proliferation of colon stem cells relative to lipids from grape derived EVs [19,48].

III. Regulate the homeostasis of intestinal immune environment.

Broccoli derived EVs regulate intestinal immune homeostasis, mainly by targeting dendritic cells (DCs). Adenosine monophosphate activated protein kinase (AMPK) in DCs is a key enzyme and enzyme pathway involved in immune homeostasis network regulation [84]. AMPK is expressed in a variety of immune cells, including macrophages, neutrophils, dendritic cells, lymphocytes and other immune cells, regulating a variety of immune cell functions, including cytokine production, chemotaxis, cytotoxicity, apoptosis and proliferation. Broccoli derived EVs can target DCs and stimulate the activation of AMPK in DCs cells, and the activation of AMPK inhibits DCs activation and monocyte recruitment; at the same time, Sulforaphane in broccoli derived EVs induce the production of tolerant DCs cells; regulatory dendritic cells are induced by these two pathways to prevent colitis in mice [47]. This study provides a solid basis for whether edible nanovesicles that present food to the intestine carry plant species-specific antigens to induce food-specific oral tolerance. Broccoli derived EVs induce immune DCs or tolerant DCs through the epithelial barrier to maintain immune homeostasis in the intestinal tract. These results suggest that edible plant derived EVs, which are naturally targeted in colon tissue and have anti-inflammatory properties, may represent a new natural non-toxic drug delivery system, which can be easily expanded to treat patients with gastrointestinal diseases such as IBD [76].

IV. Shape gut microbiota and alter host physiology.

**Figure 4:**
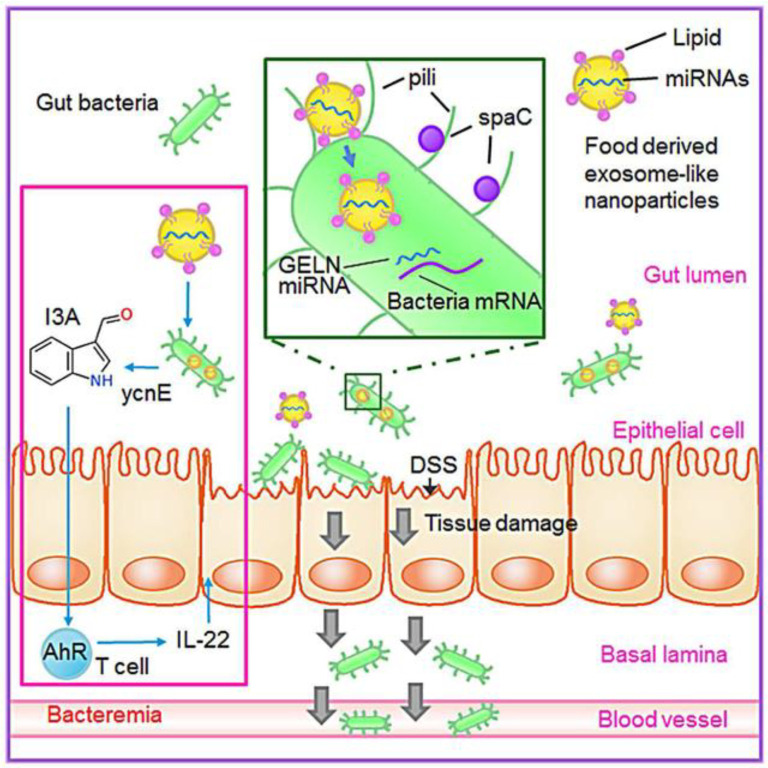
Ginger derived EVs shape gut microbiota and alter host physiology to Inhibit colitis. Reproduced with permission from ref. [85].

Improving intestinal flora is a new and effective way to improve colitis. Plant derived EVs are absorbed by intestinal microflora and contain RNA that changes the composition of microflora and host physiology. It is revealed that plant products and their effects on microbiota may be used to mitigate diseases for specific host processes. Ginger derived EVs contain miRNAs that target various genes in *Lactobacillus rhamnosus* (LGG), and then they are preferentially taken up by *Lactobacillaceae* in a ginger derived EVs lipid-dependent manner. After EVs taken by LGG, ginger derived EVs mdo-miR7267-3p-mediated targeting of the LGG monooxygenase ycnE yields increased indole-3-carboxaldehyde (I3A). RNAs or I3A of ginger derived EVs, and a ligand for the arylhydrocarbon receptor (AHR), are sufficient to induce the production of IL-22, which is linked to barrier function improvement. These functions of ginger derived EV RNAs can ameliorate mouse colitis via IL-22-dependent mechanisms [85]. The action pathway is shown in Figure 4.

#### 5.1.2. Plant Derived EVs Treatment of Alcoholic Liver

Plant derived EVs from different sources can target different specific cells, resulting in a certain curative effect. EVs mediate some key regulatory hubs, such as NRF2 and NLRP3, after entering the cells. The activation or inhibition of these hubs can affect the expression of important genes downstream. For example, ginger derived EVs target hepatocytes through blood vessels, so ginger derived EVs may have a certain therapeutic effect on liver diseases. Ginger derived EVs’ extract can protect mice from alcohol-induced liver injury. Ginger derived EVs mediate the activation of NRF2 in the liver and lead to the expression of a group of liver detoxification/antioxidant genes and inhibit the production of reactive oxygen species, which plays a role in protecting the liver to some extent. Lentinus edodes derived EVs can alleviate D-galactosamine/lipopolysaccharide-induced liver injury in mice by inhibiting the activation of NLRP3 inflammatory bodies [20]. Therefore, oral administration of these plant derived EVs may be used to treat alcoholic liver.

#### 5.1.3. The Effect of Plant Derived EVs on COVID-19

The global epidemic of COVID-19 has caused millions of deaths and seriously affected the development of the global economy, but no effective medicine has been developed against COVID-19. Plant derived EVs from dietary sources contain abundant sRNA. These sRNA have been shown to realize cross-domain regulation between plants and other species [72]. Therefore, it is possible that ginger derived EVs inhibit the effect of novel coronavirus. This possibility was further confirmed by Gopinath M. Sundaram et al. They isolated EVs from ginger and grapefruit and selected high abundance RNA for sequencing. RNA hybridization software was used to predict the target site of SARS-CoV-2 genome sequence. The experiment confirmed the existence of SARS-CoV-2 target miRNAs in ginger and grapefruit EVs [86]. However, the intracellular stability of these miRNAs and their so-called antiviral activity in vitro and in vivo need to be further studied. In addition, researchers also found the positive role of exosomes in the development of COVID-19 and the potential role of plant derived EVs in the treatment of COVID-19. In a study on the pathogenesis of COVID-19, researchers found that mice were infected with pneumonia after exposure to exosomes released by severe acute respiratory syndrome coronavirus type 2 (SARS-CoV-2)^Nsp12,Nsp13^ pulmonary epithelial cells, which proves that SARS-CoV-2 exosomes^Nsp12,Nsp13^ play an important role in the development of pulmonary inflammation. At the same time, inhaling ginger derived EVs, small RNA: Aly miR396a-5p and rlcv-miRrL1-28-3p in the lungs mediated the inhibition of *Nsp12* gene expression, respectively, which further confirmed the role of ginger derived EVs in the inhibition of SARS-CoV-2-induced cytopathic effects (CPE) [87]. In short, the cross-domain regulation of plant derived EVs and their contents RNA provides great potential for the development of plant derived EVs to treat COVID-19.

### 5.2. Plant Derived EVs as a Drug Nano-Delivery Platform for the Treatment of Diseases

Plant derived EVs, kind of tea tray-shaped bubbles wrapped in lipid bilayers that contain a variety of cargoes, can be stably loaded with small molecule drugs or nucleic acid drugs to prevent damaged degradation [58]. Plant derived EVs not only have good absorption in the intestine, but also deepen the penetration depth of drugs on the skin surface and greatly increase the amounts of drugs absorbed by the cortex [88]. Some of them can enter the brain through the blood–brain barrier through nasal administration. Some plant derived EVs, such as grape derived EVs, cannot pass through the placental barrier to protect fetal mice from drug damage when injected intravenously into pregnant mice [61]. Plant derived EVs not only have high biocompatibility, low immunogenicity and few side effects, but they are also less toxic than nanoparticles made from synthetic lipids. These facts indicate that plant derived EVs are a safe drug delivery carrier. The delivery of drugs into EVs has been confirmed by researchers. At present, the carriers used to load drugs are generally plant derived EVs or nanoparticles prepared by extraction of lipid components from plant derived EVs, and most of the drugs loaded are small molecular chemical drugs and nucleic acid drugs [89].

#### 5.2.1. Plant Derived EVs Deliver Small Molecular Chemical Drugs

Plant derived EVs can be absorbed in the intestines through oral administration, and they have different targeting due to different sources [90]. In addition, they can enter the brain through the blood–brain barrier by nasal delivery and can also be intercepted by the placental barrier, which shows a strong advantage in drug delivery. Grapefruit derived EVs can transport chemotherapeutic drugs, siRNA, DNA expression vectors and proteins to different types of cells. Researchers have significantly improved the targeting efficiency of cells expressing folate receptors by co-delivering therapeutic agents with grapefruit derived EVs and folic acid. In two kinds of tumor animal models, the therapeutic potential of grapefruit derived EVs was further proved by enhancing the inhibition of tumor growth by chemotherapy. Grapefruit derived EVs are less toxic than nanoparticles made from synthetic lipids and do not pass through the placental barrier when injected intravenously into pregnant mice, suggesting that they may be a useful tool for drug delivery [61]. The inherent biocompatibility and biodegradability of grape derived EVs with intestinal macrophages, their stability in a wide range of pH and targeting enable researchers to further develop a new oral drug delivery system based on grapefruit derived EVs. When MTX, an anti-inflammatory drug, was added to grapefruit derived EVs, the toxicity of MTX-grapefruit derived EVs in mice was significantly lower than that of free MTX, and the therapeutic effect of MTX-grapefruit derived EVs on DSS-induced colitis in mice was significantly enhanced. These findings suggest that grapefruit derived EVs can be used as an intestinal immunomodulator to maintain the homeostasis of intestinal macrophages and can be developed for oral administration of small molecular drugs to reduce the inflammatory response of human diseases [29]. Ginger derived EVs enhance targeting by combining folic acid, and the combination loaded with doxorubicin (Dox) can be effectively ingested by intestinal cancer cells. Compared with free drugs, the combination enhanced the inhibitory effect on tumors and showed good biocompatibility in the range of 200 μmol/L [40]. It was also found that ginger derived EVs can efficiently load Dox and has better pH-dependent drug release characteristics than commercial liposome Dox [83].

#### 5.2.2. Plant Derived EVs Deliver Nucleic Acids

Plant derived EVs can not only deliver small molecule drugs, but also target nucleic acids. Grapefruit derived EVs can be loaded with miR17 with tumor therapeutic effects to treat brain tumors in mice and delay the growth of brain tumors. It has been found that EVs can reach the brain through the blood–brain barrier by nasal administration, so the miR17 they carry can also enter the brain and play an inhibitory role. At the same time, EVs combined with folic acid enhanced the targeting of cytomegalovirus to folate receptor positive GL-26 brain tumors to produce better results. This strategy may provide a non-invasive treatment for brain-related diseases through nasal administration [91]. Multiple types of plant derived EVs lead to a variety of delivery pathways. Acerola derived EVs orally delivered sRNA to the digestive system in vivo. The target gene-suppressing effect in the small intestine and liver peaked 1 day after administration, indicating the potential for use as an oral DDS for nucleic acid in the digestive system [92]. Ginger derived EVs loaded with siRNA-CD98 can effectively target colon tissue and reduce the expression of CD98 by oral administration [93]. These studies show that plant derived EVs can be used as a loading platform for small molecular drugs to develop more applications of small molecular drugs.

## 6. Cell Internalization and Biodistribution of Plant Derived EVs

In cellular experiments on plant derived EVs, they can be absorbed by a variety of different animal cells. When cabbage derived EVs were incubated with human HaCaT cells at 25 °C for 15 min, fluorescence showed that cabbage derived EVs were absorbed into the cells [54]. Apple derived EVs were co-incubated with caco-2 cells and internalized into the cells within 6 h [32]. Similarly, the galactose groups on the surface of tea derived EVs can be specifically internalized by macrophages through endocytosis mediated by galactose receptors [94]. At the same time, the researchers made a preliminary study on the internalization mechanism of plant derived EVs. The internalization of plant derived EVs is closely related to the proteins on its surface. Garlic derived EVs were digested with trypsin to eliminate all proteins on its surface, which resulted in a decrease in the number of garlic derived EVs uptake by HepG2cells at the same time. The proteins responsible for this effect have been shown to include CD68 and II lectin, a protein that binds specifically to mannose [95]. To further investigate the internalization mechanism of *Asparagus cochinchinensis* derived EVs, HepG2 cells were incubated with endocytosis inhibitors (chlorpromazine hydrochloride, amiloride, nystatin, and cytochalasin D), the uptake of *Asparagus cochinchinensis* derived EVs was significantly inhibited by cytochalasin D, an inhibitor of actin polymerization required for phagocytosis, which suggested that *Asparagus cochinchinensis* derived EVs were most possibly internalized via phagocytosis pathway [68,96]. In addition, EVs from different plants show different affinity to different organs and tissues after entering mammals. Broccoli derived EVs regulate intestinal immune homeostasis by targeting DCs [47]. Ginger derived EVs showed effective colon targeting after oral administration [40]. Ginger derived EVs remained effectively in the stomach, ileum and colon 12 h after oral administration [93]. Because tea derived EVs contain natural galactose groups, they accumulate in liver tissue through galactose-mediated receptor targeting and uptake. The distribution of plant derived EVs in vivo can be related to their particle size, surface charge, and the type and number of membrane proteins [94].

## 7. Toxicity and Immunogenicity of Plant Derived EVs

Plant derived EVs are natural nanovesicles, which come from cells, so the various components in the contents of vesicles also come from cells. Under this premise, previous studies have shown that its toxicity is very low [40]. For example, when cabbage derived EVs were co-incubated with human and mouse derived HaCaT, HDF and RAW264.7 cells for 72 h, there was no significant decrease in cell survival rate, but the promotion of cell proliferation was noticed [54]. Human bone marrow mesenchymal stem cells can internalize strawberry derived EVs, and incubation for 120 h will not have a negative effect. Bone marrow mesenchymal stem cells pretreated with strawberry derived EVs can also resist oxidative stress caused by hydrogen peroxide and have potentially beneficial activity [31]. Due to its low toxicity, plant derived EVs are expected to become a widely used and safe transport carrier.

## 8. Discussion

Although plant derived EVs have positive prospects for drug delivery, there are still many limiting factors that hinder their application. Research on plant derived EVs is still in a relatively primary stage. Firstly, what are they? Although plant derived EVs are widely recognized as cell-produced bilayer membrane nanovesicles containing a range of proteins, nucleic acids, lipids, they are also determined by measuring particle size, Zeta potential and electron microscopy. However, we have not yet had a unified standard for the common or specific components of all plant derived EVs. Like exosomes, characteristic markers should be found by study [58]. Moreover, in terms of purity, the extracted plant derived EVs are difficult classify as a completely single vesicle aggregate due to the similarities between nanoparticle particle sizes. The type of purity that can be considered a single plant derived EVs is ambiguous, so the positive nature of some studies in efficacy deserves discussion. If applied in clinical practice in the future, the purity requirements for plant derived EVs will be more stringent. Secondly, the question remains as to how they are obtained. As far as the extraction of plant derived EVs are concerned, although there are diverse methods to isolate plant derived EVs, the current procedures of extracting and isolating plant derived EVs are not very efficient. Differential ultracentrifugation is not suitable for large-scale production, and the effectiveness and stability of other extraction methods need to be further investigated. Therefore, if plant derived EVs are to be produced on a large scale in the future, the current extraction and separation methods need to be further optimized. It is possible to use the SEC to realize the mass production of plant derived EVs. This method of separating and purifying particles in regard to their size can separate the EVs from the proteins of the cells themselves, which are most prone to interference, and the chromatographic column can be magnified to a certain extent according to the size. In this way, a large number of plant derived EVs may be obtained. However, with in-depth study of plant derived EVs, they will achieve mass production. Thirdly, what can they do? Most studies have shown that plant derived EVs have great potential in delivering drugs, and if they are applied in a clinical setting, problems arise. There are many delivery mechanisms, and the transport components associated with targeted delivery should be clarified. For example, studies have shown that phosphatidic acid is involved in the duration and amount of EVs accumulation in the intestine. Phosphatidylcholine promotes the migration of EVs from the intestines to the liver [34]. The physical properties, chemical composition and pharmacodynamic relationship of plant derived EVs are also a question worth studying. In short, the application of plant derived EVs has many disadvantages and the road to achieving successful application is long.

## 9. Conclusions

Plant derived EVs are important information transmitters between cells, which can change the phenotypic and functional expression of downstream cells by transporting proteins, bioactive lipids, genetic materials or bioactive components. Moreover, plant derived EVs can not only transmit information between plants of the same species, but also exchange information between mammals across kingdoms. Its internal nucleic acids, proteins, lipids and sugars also play a regulatory role in other species. Plant derived EVs show a variety of biological activities in scientific research, such as anti-inflammation, anti-cancer, anti-oxidation, antibacterial and so on. In addition, due to its own source of edible plants, it shows excellent biocompatibility, biodegradability, stability in a wide range of pH, is cheap and easy to obtain and so on. According to the above summary, the therapeutic effects of plant derived EVs targeting intestinal cells and remaining active in colitis has been further developed. Plant derived EVs, such as lemon, are specific to tumors and inducing tumor cells apoptosis, which also have superior performance in the treatment of cancer. In addition, the accumulation of plant derived EVs in the liver has a good protective effect on alcohol-induced liver and kidney injury. After the comparison of RNA sequences in plant derived EVs with those of COVID-19 by RNA program analysis, plant derived EVs may also have potential therapeutic effects on COVID-19. In addition, according to the results that plant derived EVs can enter the brain through the blood–brain barrier to inhibit tumors, plant derived EVs may have good future performance in the treatment of brain diseases. The administration routes in existing studies include oral administration, intravenous administration, transdermal administration and nasal administration; plant derived EVs can be applied to various appropriate routes of administration according to the needs of disease treatment, without the limitation of single administration. These characteristics lay the foundation for their application as a safe and effective therapeutic drug and delivery system. Researchers also studied whether plant derived EVs can be used as a drug delivery system, showing that the targeting and biocompatibility of plant derived EVs also have a good effect in the delivery of small molecular chemical drugs, nucleic acid drugs, and can not only reduce the side effects of drugs, but also improve their bioavailability. Plant derived EVs from a wide range of sources have special subcellular structures, which are conducive to cellular uptake. They can use the advantages of structural stability to transport small molecule compounds and RNA and become the carrier of anticancer drugs. In summary, as a kind of safe and effective nano-vesicles, plant extracellular vesicles are abundant, safe and effective. Plant derived EVs will have great potential in the treatment of diseases or drug delivery in the future.

## Figures and Tables

**Table 1:** The Difference between Animal Exosomes and Plant derived EVs in Size, Proteins, Lipids, Nucleic Acids.

Source	Size (nm)	Proteins	Lipids	Nucleic Acids
Animal exosomes	30~150	Targeted fusion protein Heat shock protein Membrane transporter ALIX, TSG101 CD9, CD63	Cholesterol Sphingomyelin Glycosphingolipid Ceramides	mRNA miRNAs IncRNAs
Plant derived EVs	50~1000	Actin Proteolytic enzyme Aquaporin Reticulin heavy chain Heat shock proteins	Digalactosyldiacylglycerol (DGDG) Phosphatidylglycolamine (PE) Phosphatidylglycolamine (PE) Phosphatidic acid (PA)	miRNAs

**Table 2:** Extraction of Plant derived EVs and Their Advantages and Disadvantages.

Methods	Advantages	Disadvantages
UC	High purity, Simple operation	Long time, High cost
PEG precipitation	Simple operation, Economical, Rapid rate	Low purity, Low yield
SEC	High purity, High yield, Maintaining good biological function	Long time, A heavy workload
Electrophoresis	Convenient, High yield, low cost	Low purity, Low temperature condition
Test kit	Sample, Convenient	Low purity, Low yield, Small-scale

**Table 3:** The Bioactivity and Action Pathway of Multiple Plant derived EVs.

Bioactivity	Source	Action Pathway
Anti-inflammatory	Ginger	Gene regulation
	Grapefruit	Macrophage
	Grape	Intestinal flora
Antitumor	Lemon	Induced apoptosis
	Ginger	
	Grapefruit	
	*Asparagus cochinchinensis*	
	Tea	
Antibiosis	Coconut	Gene silencing
	*Arabidopsis thaliana*	
	Ginger	
Antibiosis	Lemon	Dependent on antioxidant components in Plant–derived EVs, such as VC
	Apple	
	Strawberry	
	Tea	

**Table 4:** Administration and Inhibitive Diseases of Different Plant derived EVs.

EVs Source	Route of Administration	Disease
Grapefruit	Oral	Colitis
	Nasal	Brain Tumor
Grape	Oral	Colitis
	iv.	\
Broccoli	Oral	Colitis
Ginger	Oral	Colitis, Alcoholic liver
Cucumber	Transdermal	\
Tea flower	Oral, iv.	Breast cancer
Tea	Oral	Colitis

## Data Availability

Not applicable.
